# Intestinal Microbiota of Older Japanese Females Adhering to a Traditional Japanese Brown Rice-Based Diet Pattern

**DOI:** 10.3390/nu18020219

**Published:** 2026-01-09

**Authors:** Kouta Hatayama, Aya Ebara, Chihiro Hirano, Kanako Kono, Hiroaki Masuyama, Iyoko Ashikari

**Affiliations:** 1 Symbiosis Solutions Inc., Tokyo 101-0064, Japan; 2 Ashikari Clinic, Tokyo 164-0011, Japan

**Keywords:** intestinal microbiota, gut microbiota, Shokuyo diet, traditional Japanese diet, brown rice, Japanese older adults, dietary patterns

## Abstract

**Background/Objectives**: Some Japanese people still adhere to a systematic traditional Japanese diet pattern (the Shokuyo diet) consisting mainly of brown rice, vegetables, legumes, and small amounts of fish. We investigated the impact of this dietary pattern on the intestinal microbiota of its female consumers. **Methods**: The intestinal microbiota of 19 Japanese females in their 60s and 70s consuming the Shokuyo diet (Shokuyo diet group) and 50 females of the same age consuming a normal Japanese diet (NJ diet group) were compared. The NJ diet group was further subdivided into a healthy NJ diet H subgroup, comprising females (*n* = 19) without any diseases, and an unhealthy NJ diet UH subgroup (*n* = 31) consisting of females with certain diseases, and a subgroup analysis was performed. Intestinal microbiota analysis was performed using 16S rRNA gene amplicon sequencing. **Results**: The β-diversity of the intestinal microbiota significantly differed between the Shokuyo diet and NJ diet groups. Similarly, in the subgroup analysis, β-diversity also significantly differed between the NJ diet UH subgroup and the Shokuyo diet group. However, no significant difference was observed between the NJ diet H and Shokuyo diet groups. These results indicate that the intestinal microbial composition of the Shokuyo diet group resembled that of the healthy participants, and that differences in intestinal microbial composition between the Shokuyo and NJ diet groups were strongly influenced by the presence of participants with diseases in the NJ diet group. That is, differences in β-diversity may have been strongly mediated by the health status of the participants. **Conclusions**: Consumption of the Shokuyo diet may be associated with a healthy intestinal microbial composition in older Japanese female, suggesting its potential as a viable dietary intervention option.

## 1. Introduction

Japan is among the countries with the longest life expectancy, especially that of females, and a rapidly aging population [1]. However, beyond simply extending lifespan, prolonging the number of years lived in good health is desirable for individuals and, ultimately, for society as a whole.

Dysbiosis of the intestinal microbiota is associated with the onset and progression of numerous diseases, including obesity, type 2 diabetes, inflammatory bowel disease, irritable bowel syndrome, colorectal cancer, cardiovascular disease, and cognitive decline [2,3]. Increasing evidence suggests that maintaining a good balance of the intestinal microbiota and avoiding dysbiosis is important for healthy aging. However, even if intestinal microbial dysbiosis occurs, restoring a healthy microbiota can support a healthy and long life [3].

Intestinal microbial dysbiosis can be improved through various methods, including the consumption of prebiotics, which are food components that promote the growth and function of specific beneficial intestinal microorganisms; probiotics, which are live microorganisms that confer health benefits to the host when ingested in appropriate amounts; and postbiotics, which are inactivated microbial cells mixed with or without metabolic products or cellular components that provide health benefits to the host. Other methods include fecal microbial transplantation, which is the transfer of fecal microbiota from a healthy individual to the intestines of a patient, and dietary interventions [4,5]. Among these, dietary intervention targeting daily meals holds the potential to fundamentally reshape the composition of the intestinal microbiota and, theoretically, promote broader shifts toward a healthier microbial composition [3]. These dietary inventions include the Mediterranean diet (MedDiet), which is characterized by increased consumption of vegetables, legumes, fruits, nuts, olive oil, and fish, as well as low consumption of red meat, dairy products, and saturated fats. A one-year MedDiet intervention study targeting older adults in five European countries, namely the United Kingdom, France, the Netherlands, Italy, and Poland, suggested the potential of this diet for promoting healthy aging [6]. However, the MedDiet is unfamiliar to Japanese people, who have a distinct food culture, and older Japanese people may find it especially difficult to adhere to this diet.

For Japanese people, especially older adults, dietary interventions should incorporate dietary patterns aligned with traditional Japanese food culture. Such a candidate dietary pattern suitable for Japanese people is the Shokuyo diet, which is a traditional Japanese diet centered on brown rice, vegetables, legumes, and small amounts of fish, recommended by the non-profit organization Japan Association of Holistic Medicine [7]. The Shokuyo diet recommends moderate food intake, and in addition to the foods mentioned above, it includes abundant consumption of fermented foods and seaweed, resulting in a dietary pattern rich in dietary fiber (an example meal: brown rice topped with ground sesame seeds, miso soup with root vegetables and seaweed, one side dish, Japanese pickles). Similarly to the MedDiet, the Shokuyo diet also emphasizes the consumption of vegetables, legumes, and small amounts of fish. It is based on the concept of “Shokuyo,” proposed by 19th-century medical doctor Sagen Ishizuka. “Shokuyo” is a traditional Japanese dietary philosophy that emphasizes harmony between food and health, aiming to maintain or restore well-being through natural and balanced nutrition [8]. The Japan Association of Holistic Medicine has systematized the Shokuyo diet, compiling a collection of recipes, and actively conducts educational and outreach activities on its philosophy, knowledge, and practical methods. Consequently, a foundation has been established, enabling many people to practice the Shokuyo diet. Based on these findings, we hypothesized that the Shokuyo diet may be associated with a healthy intestinal microbiota, as is the case with the MedDiet. More specifically, we anticipated that individuals adhering to the Shokuyo diet would have an intestinal microbiota rich in beneficial bacteria such as short-chain fatty acid-producing bacteria and low in harmful bacteria associated with disease. However, the effect of this diet on the composition of the intestinal microbiota of individuals consuming it and its association with health maintenance and promotion remain unclear.

Therefore, the present study aimed to clarify the characteristics of the intestinal microbiota in Japanese older adults who have been following the Shokuyo diet for a long time. Specifically, we investigated whether the intestinal microbiota induced by the Shokuyo diet is associated with the maintenance and improvement of overall health to determine its potential as a dietary option to prevent or improve intestinal microbial dysbiosis in Japanese older adults. We initially planned to analyze data by sex, reflecting potential sex-based differences in the intestinal microbiota [9]. However, as most of the participants recruited were females in their 60s and 70s, our final analysis was limited to this group.

## 2. Materials and Methods

### 2.1. Participants in the Shokuyo Diet Group

Participants adhering to the Shokuyo diet were recruited through the Japan Association of Holistic Medicine from August to November 2021, with 19 male and 35 female participants. Using a questionnaire completed at the time of participation, participants reported the duration of their Shokuyo diet and frequency of brown rice consumption. Background information on participants was collected through self-reported questionnaires administered at the same time as stool sample collection. This information included age, sex, height, weight, pregnancy and breastfeeding status, use of antibiotics and enemas, and disease status.

Inclusion criteria were as follows: (1) Japanese, (2) primarily living in Japan, (3) no long-term overseas residence in the past year, (4) aged 20 years or older, (5) incorporating Shokuyo diet into daily life, and (6) member of the Japan Association of Holistic Medicine. Exclusion criteria were as follows: (1) incomplete intestinal microbiota data, (2) incomplete questionnaire responses, (3) non-Japanese, (4) antibiotic use within the past 3 months, (5) enema or suppository use, (6) colostomy, (7) pregnancy or breastfeeding, (8) continuous adherence to Shokuyo diet for less than one year, and (9) consumption of brown rice less than three days per week. Participants with low adherence to the Shokuyo diet were excluded based on the latter two exclusion criteria (8 and 9).

**Figure 1:**
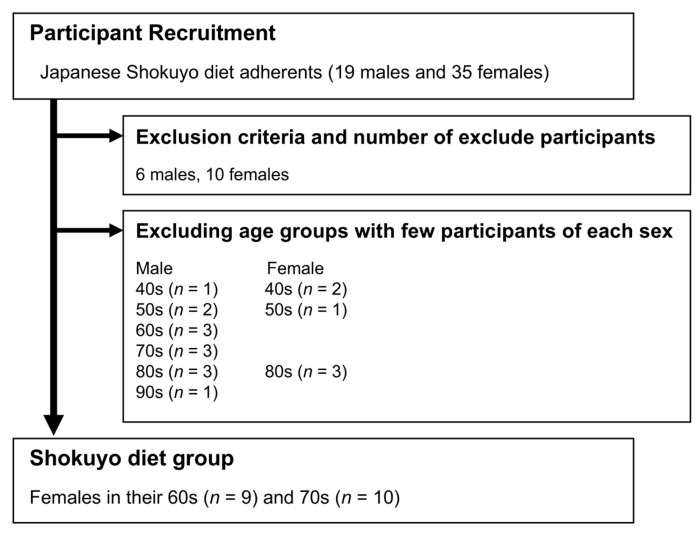
Selection of the Shokuyo diet groups from the recruited participants.

Based on these inclusion and exclusion criteria, the main participants adhering to the Shokuyo diet were females in their 60s and 70s (Figure 1). Considering that the intestinal microbiota is affected by sex and age [9], this study focused on females in their 60s and 70s, who were assigned to the Shokuyo diet group (19 females).

### 2.2. Participants in the Control Group

Using the Symbiosis Microbiome Analysis Database (SymMAD; data not publicly available for privacy reasons), a large database of intestinal microbiota collected from Japanese individuals and owned by Symbiosis Solutions Inc. (Tokyo, Japan) [9], data were extracted from females aged 60–79 years who consumed a diet typical of the contemporary Japanese diet. These participants were designated as the Normal Japanese diet (NJ diet) group (*n* = 50), and their data were collected between April 2020 and May 2024, via the intestinal microbiota testing service of the company. The typical diet for the current Japanese population was defined as one whose daily energy intake falls within the 25th–75th percentile of energy intake reported in the National Health and Nutrition Survey 2019 [10] of Japan (approximately 60–64 years: 1431–2011 kcal; 65–75 years: 1496–2057 kcal; 75–79 years: 1356–1914 kcal). Energy intake of the NJ diet group was calculated using the food frequency questionnaire based on food groups version 6 and Excel Eiyokun Version 9.0 (Kenpakusha, Tokyo, Japan).

The inclusion criteria for the NJ diet group were as follows: (1) Japanese; (2) primary residence in Japan; (3) no long-term overseas residence in the past year; (4) aged 60–79 years; (5) female; and (6) consumption of the contemporary Japanese diet. The exclusion criteria were as follows: (1) incomplete intestinal microbiota data, (2) incomplete questionnaire responses, (3) non-Japanese, (4) antibiotic use within the past 3 months, (5) enema or suppository use, (6) colostomy, and (7) pregnancy or breastfeeding.

### 2.3. Intestinal Microbial Analysis

Stool sample collection, DNA extraction, and 16S rRNA gene sequence (variable regions V3–V4) analysis were conducted using the MiSeq system (MiSeq Software v4.1.0) (Illumina Inc., San Diego, CA, USA), as previously described [11]. The taxonomic affiliation of amplicon sequence variants was determined using the Ribosomal Database Project training set, version 18 [12]. Analyses of α-diversity (Shannon index, Simpson index, number of taxa, and Pielou index) and group-wise comparisons of the intestinal microbiota were conducted using the analysis of variance (ANOVA)-like differential expression tool (ALDEx2) [13,14,15], as described by Hatayama et al. [16]. The centered log ratio-transformed intestinal microbiota data were used for comparisons with ALDEx2. To visualize β-diversity, non-metric multidimensional scaling (NMDS) based on the Bray–Curtis index was performed using the metaMDS function in the R vegan package (2.6-4) of R (v. 4.2.0; The R Foundation Group, Vienna, Austria) [17] was used for NMDS. Permutational Multivariate Analysis of Variance (PERMANOVA) was performed using the adonis function in the same package, with 9999 permutations. Permutational multivariate analysis of dispersion (PERMDISP) (multivariate homogeneity of group dispersions) [18] was performed using the betadisper function in the same package.

### 2.4. Statistical Analysis

Statistical analysis was conducted using R software (v. 4.2.0). The Wilcoxon rank-sum test was used for intergroup comparisons of age, height, weight, body mass index (BMI), and alpha diversity indices. Fisher’s exact test was used to compare the number of participants with diseases. Statistical significance was set at *p* < 0.05.

## 3. Results

### 3.1. Shokuyo and NJ Diet Groups

A total of 19 female participants aged between 60 and 79 years, who adhered to the Shokuyo diet long-term, were assigned to the Shokuyo diet group. A total of 50 female individuals of the same age who typically consumed a normal Japanese diet were assigned to the NJ diet group. There were no significant differences in age between the Shokuyo diet and NJ diet groups (Table 1). However, there were significant differences in weight and BMI between the groups, whereas we observed no significant differences in height between them. Weight and BMI may be associated with intestinal microbiota, but they change in response to diet. Therefore, this study did not treat them as confounding factors in the diet-intestinal microbiota association. In this study, the following confounding factors were controlled for between the two groups: ethnicity (Japanese), residential region (Japan), sex (female), and age (60–79 years).

### 3.2. Comparison of Intestinal Microbiota Between the Shokuyo and NJ Diet Groups

**Figure 2:**
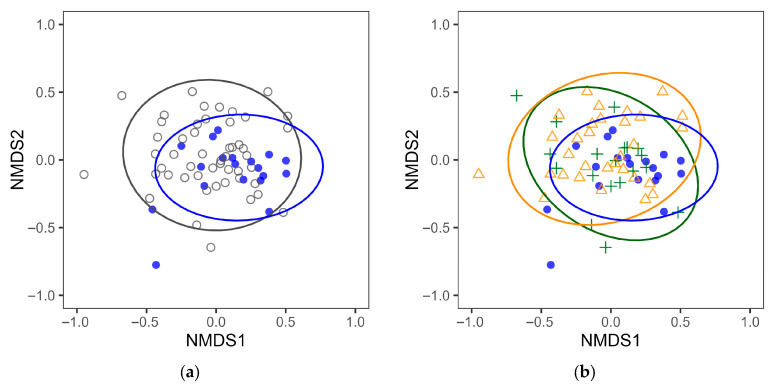
Non-metric multidimensional scaling (NMDS) plotting based on the Bray–Curtis index of intestinal microbiota. (**a**) Plots for the Shokuyo (blue closed circle) and NJ diet (gray open circle) groups. (**b**) Plots for the Shokuyo diet group (blue closed circle), NJ diet H subgroup (green cross), and NJ diet UH subgroup (orange open triangle). Ellipses in NMDS indicate 95% confidence intervals around the centroid of each group. The same scale was used for (**a**,**b**) (Stress: 0.2014948).

To clarify the effects of differences in daily diet on the intestinal microbiota, we compared the intestinal microbiota of individuals in the Shokuyo and NJ diet groups. At the genus level, we observed no significant differences in α-diversity indices, including the Shannon index, Simpson index, number of taxa, and Pielou index, of the intestinal microbiota between the two groups (Table 1). In contrast, the β-diversity of the intestinal microbiota significantly differed between the two groups. Specifically, while there was no significant difference in the homogeneity test of variance, PERMANOVA revealed a significant difference (PERMDISP *p* = 0.329, PERMANOVA *p* = 0.031). Moreover, we noted a tendency toward differences in the NMDS plot positions between the two groups (Figure 2a; note that the stress value exceeds 0.2, indicating a poor representation). Therefore, β-diversity analysis revealed differences in the intestinal microbial composition between the Shokuyo diet and NJ diet groups.

**Figure 3:**
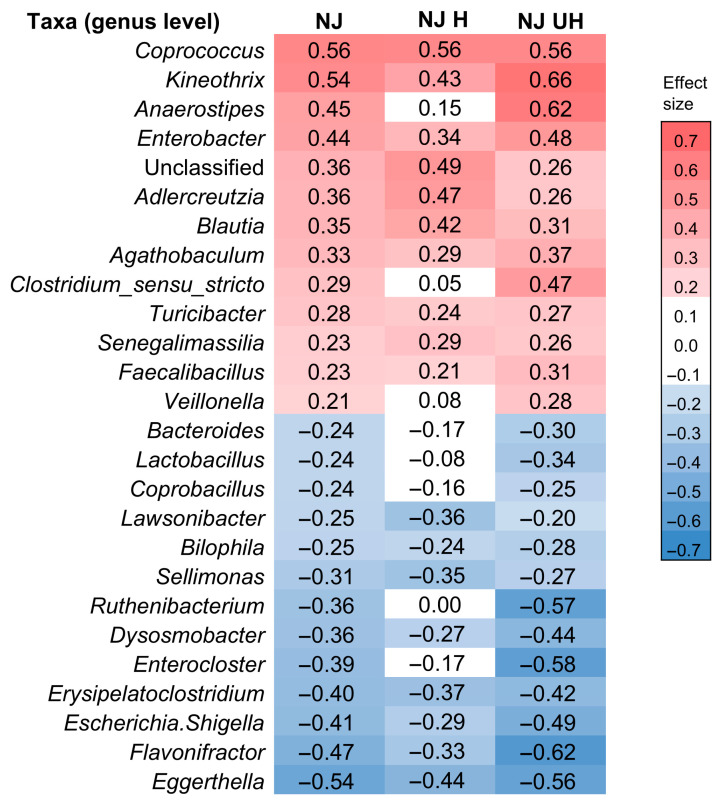
Effect size of ALDEx2 analysis. NJ presents the comparison of intestinal microbiota between the Shokuyo and NJ diet groups, NJ H depicts the comparison between the Shokuyo diet group and the NJ diet H subgroup. NJ UH presents the comparison between the Shokuyo diet group and the NJ diet UH subgroup. Taxa with effect sizes greater than 0.2 (red) and less than −0.2 (blue) indicate that they are more abundant and less abundant in the Shokuyo diet group than in the control group, respectively. However, note that no significant differences were observed in the centered log ratio-transformed data for any of the taxa, except for *Kineothrix* in NJ UH (Wilcoxon rank-sum test with Benjamini-Hochberg correction).

We further analyzed differences in intestinal bacteria taxa between the Shokuyo and NJ diet groups based on the effect size of ALDEx2, which measures the effect independently of sample size. Therefore, this indicator is particularly useful for identifying differences in intestinal bacteria between two groups with limited sample sizes. In this study, we considered bacterial taxa (genus level) with an effect size greater than 0.2 as more abundant in the Shokuyo diet group than in the NJ diet group, and those with an effect size below −0.2 as less abundant in the Shokuyo diet group. The effect size criteria (absolute values) was set based on the criteria proposed by Cohen [19] (considering values of 0.2 or higher as meaningful). We identified a total of 13 more abundant taxa and 13 less abundant taxa in the Shokuyo diet group than in the NJ diet group (Figure 3).

### 3.3. Subgroup Analysis Based on Disease Status

Both the Shokuyo and NJ diet groups included healthy participants without any disease and participants with certain diseases. The complete list of these diseases is presented in Supplementary Table S1. We observed no significant differences in the percentages of participants with diseases between the two groups (Table 2). However, compared to the NJ diet group, the Shokuyo diet group included a lower proportion of participants with each disease, except for pollinosis and dyslipidemia. No participants in the Shokuyo diet group had bone and joint diseases, dizziness, obesity, type 2 diabetes, constipation, or insomnia.

Overall, these comparative results indicate that the composition of the intestinal microbiota of individuals in the Shokuyo diet group differed from that of participants in the NJ diet group. However, our analysis of participants with diseases in the two groups did not clarify whether the composition of the intestinal microbiota in the Shokuyo diet group was associated with health or disease states. Therefore, we performed an additional analysis, subdividing the NJ diet group into an NJ diet H subgroup (*n* = 19), consisting of healthy participants without any diseases, and an NJ diet UH subgroup (*n* = 31), comprising participants with a given disease. We then assessed the similarity of their intestinal microbiota through comparisons with the Shokuyo diet group. Comparisons between each NJ diet subgroup and the Shokuyo diet group revealed significant differences in weight and BMI, but not in height or the α-diversity indices of the intestinal microbiota (Supplementary Tables S2 and S3). We also observed a significant difference in age between the Shokuyo diet group and the NJ diet H subgroup, but not between the Shokuyo diet group and the NJ diet UH subgroup.

In NMDS visualizing the β-diversity of the intestinal microbiota, the plots of the Shokuyo diet group and NJ diet H subgroup exhibited relatively similar positions (Figure 2b). In contrast, the NJ diet UH subgroup plots exhibited different positions from the Shokuyo diet group. Moreover, PERMANOVA results revealed no significant differences between the Shokuyo diet group and NJ diet H subgroup (PERMANOVA *p* = 0.072, PERMDISP *p* = 0.480), whereas significant differences emerged between the Shokuyo diet group and NJ diet UH subgroup (PERMANOVA *p* = 0.023, PERMDISP *p* = 0.520). These results indicate that the intestinal microbial composition of individuals in the Shokuyo diet group was not significantly different from that of participants in the NJ diet H subgroup, whereas it significantly differed from that of individuals in the NJ diet UH subgroup. These results suggest that the intestinal microbial composition of individuals in the Shokuyo diet group may be associated with health (free from diseases).

Effect size is useful for comparing results from analyses with different sample sizes, as it represents a measure of the standardized degree of effect. ALDEx2 analysis (effect size) revealed 36 significantly different intestinal bacteria taxa between the NJ diet UH subgroup and the Shokuyo diet group (Supplementary Table S4). Notably, these 36 taxa included 26 taxa that differed between the NJ diet and Shokuyo diet groups (Figure 3). In contrast, 30 taxa differed between the NJ diet H subgroup and the Shokuyo diet group (Supplementary Table S5). Of the 26 taxa that differed between the Shokuyo diet and NJ diet groups, 8 were not included (Figure 3): *Anaerostipes*, *Clostridium*_*sensu*_*stricto*, *Veillonella*, *Bacteroides*, *Lactobacillus*, *Coprobacillus*, *Ruthenibacterium*, and *Enterocloster*. These results indicate that the 26 taxa that differed between the NJ diet and Shokuyo diet groups were strongly influenced by differences between the NJ diet UH subgroup and the Shokuyo diet group, likely owing to the presence of diseases.

## 4. Discussion

In this study, we analyzed the intestinal microbiota of Japanese female participants in their 60s and 70s who had long adhered to the Shokuyo diet. Our analysis focusing on differences in daily dietary patterns indicated that the β-diversity of the intestinal microbiota of participants in the Shokuyo diet group differed from that of participants in the NJ diet group. Furthermore, our subgroup analysis focusing on health status revealed that the β-diversity of the intestinal microbiota of participants in the Shokuyo diet group was not significantly different from that of participants in the NJ diet H subgroups (without disease), whereas it was significantly different from that of participants in the NJ diet UH subgroups (with certain diseases). We also identified 26 bacterial taxa that differed between the Shokuyo and NJ diet groups, which were considered to be strongly influenced by the differences between the Shokuyo diet group and the NJ diet UH subgroup. These results indicate that the differences in the β-diversity of the intestinal microbiota observed between the Shokuyo and NJ diet groups may have been strongly influenced by participants with diseases included in the NJ diet group.

The ALDEx2 effect size analysis between the Shokuyo diet group and the NJ diet UH subgroup revealed that *Dysosmobacter*, *Eggerthella*, *Enterocloster*, *Escherichia*, *Shigella*, *Erysipelatoclostridium*, *Flavonifractor*, and *Ruthenibacterium* exhibited values below −0.4 (Figure 3 and Supplementary Table S4), indicating that these taxa were particularly less abundant in the Shokuyo diet group. The relative abundances of *Dysosmobacter*, *Eggerthella*, *Escherichia*, *Shigella*, *Erysipelatoclostridium*, and *Flavonifractor* were lower in the Shokuyo diet group, even when compared to the NJ diet H subgroup. Therefore, the Shokuyo diet may be more effective than an NJ diet in suppressing these bacterial taxa.

According to previous reports, *Eggerthella*, which can oxidize bile acid, and *Erysipelatoclostridium*, which can degrade IgA, may be associated with intestinal microbial dysbiosis [16,20,21,22,23]. *Enterocloster* is associated with type 2 diabetes, fatty liver disease, autism, and nonalcoholic steatohepatitis [24,25,26,27]; *Escherichia* and *Shigella* may potentially be pathogenic. *Flavonifractor* and *Ruthenibacterium*, along with *Eggerthella* and *Erysipelatoclostridium*, are potentially associated with mild cognitive impairment in Japanese females [16]. Based on these findings, it is hypothesized that the low relative abundance of *Eggerthella*, *Enterocloster*, *Erysipelatoclostridium*, *Escherichia*, *Shigella*, *Flavonifractor*, and *Ruthenibacterium* may be associated with health benefits. In contrast, *Dysosmobacter* has been reported to potentially be associated with preventing diet-induced obesity and improvement of glucose tolerance [28,29], but its relative abundance was low in the Shokuyo diet group.

In the present study, compared to the NJ diet UH subgroup, the levels of *Anaerostipes*, *Coprococcus*, *Clostridium*_*sensu*_*stricto*, *Enterobacter*, and *Kineothrix* were particularly high in the Shokuyo diet group (effect size > 0.4, Figure 3 and Supplementary Table S4). Furthermore, the Shokuyo diet may increase the abundances of *Adlercreutzia*, *Agathobaculum*, *Blautia*, *Faecalibacillus*, *Senegalimassilia*, and *Turicibacter* compared to the NJ diet H subgroups (Figure 3). Although polished rice is a staple food in an NJ diet, brown rice, which is rich in dietary fiber, vitamin B complex, and minerals, is the staple food in the Shokuyo diet. Therefore, differences in dietary patterns, including differences in staple foods, may have affected the abundance of these intestinal bacteria.

We investigated the previous reports on these taxa with high relative abundance in the Shokuyo diet group. *Anaerostipes*, *Coprococcus*, and *Kineothrix*, which produce butyric acid, are associated with maintaining host health [30,31,32]. In contrast, as *Clostridium*_*sensu*_*stricto* and *Enterobacter* include pathogenic species [33,34], their high abundance may pose potential health risks. *Adlercreutzia* is associated with equol production [35], and *Agathobaculum* can improve age-related cognitive decline [36]. *Blautia* may exert both beneficial and harmful effects on human health [37]. Moreover, *Faecalibacillus* may be associated with human health, given its low abundance in the intestinal microbiota of patients with malignancies [38]. *Senegalimassilia* may serve as a protective factor against hypertension [39], while *Turicibacter* may be associated with health by affecting host bile acid and lipid metabolism [40]. Therefore, many of these taxa have been reported to be associated with health. However, further studies are needed to clarify their relationship with the Shokuyo diet.

The β-diversity of the intestinal microbiota of individuals in the Shokuyo diet group was not significantly different from that of individuals in the NJ diet H subgroup. Therefore, adhering to the Shokuyo diet may be associated with a healthy intestinal microbial composition. However, the Shokuyo diet group included participants who had dyslipidemia, hypertension, and pollinosis; therefore, following the Shokuyo diet does not necessarily prevent the development of these diseases. Nevertheless, participants in the Shokuyo diet group did not have high BMI values and did not have type 2 diabetes, obesity, constipation, osteoporosis, dizziness, or other diseases. Overall, while the intestinal microbiota formed by the Shokuyo diet does not necessarily guarantee health maintenance, it may be associated with suppressing the onset of certain diseases and supporting health maintenance.

In this study, the NJ diet had no restrictions other than consuming energy within the specified range and not eating brown rice frequently. Therefore, this diet includes various dietary styles that are feasible in Japan today. A varied diet leads to diversification in intestinal microbial composition and health status. Therefore, the NJ diet group included both healthy participants and those with diseases, and differences in β-diversity of the intestinal microbiota related to health status were also observed in NMDS (NJ diet group plots were scattered in NMDS, Figure 2). Given the wide variety of food choices available in Japan today, choosing and consuming meals that contribute to maintaining and improving health daily can be challenging. Depending on dietary content, intestinal microbiota associated with disease may be developed and persist. In contrast, long-term adherence to the Shokuyo diet may lead to an intestinal microbial composition characteristic of this diet. In our NMDS analysis, the plots of the Shokuyo diet group were observed as a cluster, indicating that their intestinal microbiota compositions were relatively similar (Figure 2). Overall, our analysis of the intestinal microbiota of Japanese older female participants adhering to the Shokuyo diet provides reference information on the intestinal microbiota associated with adherence to this diet (the mean values, median values, and detection rates of the relative abundance of each intestinal bacterial taxon are presented in Supplementary Table S6). Such reference information on the expected intestinal microbial composition for specific dietary patterns can be useful when developing dietary interventions to improve gut health and guide dietary changes.

The composition of the intestinal microbiota is affected by various factors, including diet. The MedDiet, characterized by increased consumption of vegetables, fruits, legumes, fish, olive oil, and nuts, and reduced consumption of red meat, dairy products, and saturated fats, is one of the most studied diets. It possesses a wide range of benefits that maintain and promote health [6,41]. The Shokuyo diet is similar to the MedDiet in that it focuses on vegetables, legumes, and small amounts of fish. However, Japanese people are likely to find the Shokuyo diet more familiar and easier to accept as a daily diet than the MedDiet, as it is based on Japanese cuisine. Moreover, brown rice, the staple food of the Shokuyo diet, requires more chewing than polished rice, thus it induces satiety even after consuming small portions. Therefore, overeating is less likely to occur, which may be associated with the decreased body weight and BMI observed in the Shokuyo diet group. Brown rice is also rich in dietary fiber, which may promote butyric acid-producing bacteria. In addition, limiting fish intake to small amounts and avoiding excessive consumption of meat may reduce the production of ammonia and hydrogen sulfide, which are protein-derived intestinal bacterial metabolites that are harmful in high concentrations. Notably, a study investigated changes in the intestinal microbiota of individuals over one year and demonstrated that dietary changes during travel from developed to developing countries during the study period cause significant changes in the composition of intestinal microbiota. However, these changes were reversed within two weeks of returning from travel [42]. This suggests that while dietary intervention can alter the intestinal microbiota, discontinuing dietary intervention and returning to previous dietary patterns may cause the intestinal microbiota to return to its pre-intervention state. Therefore, dietary patterns used for intervention should be easy to continue. Notably, our study included participants who had been following the Shokuyo diet for more than 40 years (Supplementary Table S7), indicating that this dietary pattern is sustainable over time. Overall, given differences in food cultures across countries and regions, it is crucial to select dietary patterns that are appropriate for the cultural background of the participants to achieve the desired results. The Shokuyo diet is a dietary pattern adapted to Japanese food culture and would be suitable for dietary intervention for Japanese people; however, it may not be suitable for application outside Japan. Therefore, the development of dietary patterns for dietary intervention adapted to the food culture of each country and region should be widely promoted.

This study had certain limitations. First, the sample size used in this study was small, necessitating additional studies involving a larger number of participants to confirm the validity of our findings. Second, it cannot be ruled out that bias existed in the recruitment of participants. Participants in the control group are users of the intestinal microbiota testing service, and there is a difference in the recruitment period compared to participants in the Shokuyo diet group. This study compared the two groups based on the fact that both groups consisted of general females, that the adult intestinal microbiota is long-term stable [43], and that there were no major changes in Japan’s social conditions during the recruitment period. However, the impact of these structural differences on the study was not verified beforehand. Third, while this study performed group comparisons by controlling for ethnicity, residential area, sex, age, and disease status (subgroup analysis) as confounding factors, uncontrolled potential confounding factors may remain. In observational studies, confounding factors are difficult to control completely. Future validation of this study is necessary through controlled intervention studies, such as randomized controlled trials, which better control for confounding factors. Fourth, because this study was observational, causal inference is not possible. Fifth, 16S rRNA gene amplicon sequencing is used for analyzing the intestinal microbiota. This approach has limitations, such as the resolution limitations in classifying intestinal bacteria and the lack of functional information. Sixth, adherence to the Shokuyo diet does not guarantee complete disease prevention or improvement of existing health conditions, as the relationship between adherence to the Shokuyo diet and health status requires further studies. Seventh, as the composition of intestinal microbiota is associated with ethnicity, geographical location, age, and sex [9,44], the results of this study may be limited to older Japanese females. Finally, this study only investigated changes in the intestinal microbiota of older Japanese females following the Shokuyo diet, and it is unclear whether this diet could improve intestinal microbial dysbiosis.

## 5. Conclusions

Adherence to the Shokuyo diet may be associated with the healthy intestinal microbiota composition in older Japanese females. In NJ diets, selecting and maintaining meals that promote the establishment of healthy intestinal microbiota is challenging. In contrast, the Shokuyo diet is a systematic dietary pattern, and its advantage lies in being easy to follow and adhere to over time. However, the application of the Shokuyo diet as a dietary intervention requires validation in future studies to determine whether adherence to this diet improves intestinal microbial dysbiosis. Moreover, studies on the intestinal microbiota of both males and females in various age groups who adhere to the Shokuyo diet are also important to expand the range of people who can benefit from dietary therapy using the Shokuyo diet.

## Figures and Tables

**Table 1:** Comparison between the Shokuyo and NJ diet groups.

	Shokuyo Diet (*n* = 19)	NJ Diet (*n* = 50)	*p*-Value
Age (year)	70.4 ± 4.1	68.5 ± 5.6	0.156
Height (cm)	155.0 ± 4.3	155.9 ± 5.6	0.757
Weight (kg)	48.4 ± 6.8	54.2 ± 7.4	0.006
BMI (kg/m^2^)	20.0 ± 2.6	22.3 ± 2.9	0.006
α diversity indices:			
Shannon index	2.86 ± 0.24	2.87 ± 0.20	0.600
Simpson index	0.91 ± 0.02	0.91 ± 0.03	0.361
Number of taxa	54.00 ± 8.63	53.62 ± 10.65	0.702
Pielou index	0.72 ± 0.04	0.73 ± 0.04	0.444

Data show mean ± standard deviation. *p*-values obtained from the Wilcoxon rank-sum test. BMI: body mass index; NJ, normal Japanese. Statistical significance was set at *p* < 0.05.

**Table 2:** Number of participants with diseases in the Shokuyo and NJ diet groups.

Disease	Shokuyo Diet (*n* = 19)	NJ Diet (*n* = 50)	*p*-Value
With some kind of disease	8 (42.1%)	31 (62.0%)	0.177
High blood pressure	3 (15.8%)	11 (22.0%)	0.742
Dyslipidemia	4 (21.1%)	8 (16.0%)	0.725
Bone and joint diseases	0 (0.0%)	6 (12.0%)	0.177
Pollinosis	2 (10.5%)	4 (8.0%)	0.664
Dizziness	0 (0.0%)	3 (6.0%)	0.556
Obesity	0 (0.0%)	3 (6.0%)	0.556
Type 2 diabetes	0 (0.0%)	3 (6.0%)	0.556
Constipation	0 (0.0%)	3 (6.0%)	0.556
Insomnia	0 (0.0%)	3 (6.0%)	0.556

Diseases that affected at least three participants in both groups are presented. The percentage in parentheses indicates that of the group. *p*-values obtained from Fisher’s exact test. Statistical significance was set at *p* < 0.05.

## Data Availability

The data for this study were derived from the non-publicly available database (SymMAD) maintained by Symbiosis Solutions Inc. The data presented in this study are available upon request from the corresponding author. These data are not publicly available because of privacy concerns.

## Supplementary Materials

The following supporting information can be downloaded at: https://www.mdpi.com/article/10.3390/nu18020219/s1, Table S1: Diseases present in among participants in the Shokuyo and NJ diet groups; Table S2: Comparison between the Shokuyo diet group and the NJ diet H subgroup; Table S3: Comparison between the Shokuyo diet group and the NJ diet UH subgroup; Table S4: Effect sizes, relative abundance, and detection rates of each taxon in the ALDEx2 analysis between the Shokuyo diet group and the NJ diet UH subgroup; Table S5: Effect sizes, relative abundance, and detection rates of each taxon in the ALDEx2 analysis between the Shokuyo diet group and the NJ diet H subgroup; Table S6: Relative abundance and detection rate of each intestinal bacterial taxon in the Shokuyo diet group (*n* = 19); Table S7: Duration of the Shokuyo diet.

## Abbreviations

The following abbreviations are used in this manuscript:

BMI	Body mass index
MedDiet	Mediterranean diet
NJ	Normal Japanese
NMDS	Non-metric multidimensional scaling
PERMANOVA	Permutational multivariate analysis of variance
PERMDISP	Permutational multivariate analysis of dispersion
