# Supplementary material for: Intestinal Microbiota of Older Japanese Females Adhering to a Traditional Japanese Brown Rice-Based Diet Pattern
Source: Nutrients. 2026 Jan 9;18(2):219. doi: 10.3390/nu18020219 (PMC12844664; doi:10.3390/nu18020219)
Supplement: Supplementary file 1 [file nutrients-18-00219-s001.zip › nutrients-4072724-supplementary.pdf]

nutrients

MDPI

Article

Intestinal Microbiota of Older Japanese Females Adhering to a Traditional Japanese Brown Rice-Based Diet Pattern

Kouta Hatayama[1,*], Aya Ebara[1], Chihiro Hirano[1], Kanako Kono[1], Hiroaki Masuyama[1] and Iyoko Ashikari[2]

[1] Symbiosis Solutions Inc., Tokyo 101-0064, Japan
[2] Ashikari Clinic, Tokyo 164-0011, Japan
* Correspondence: Hatayama@symbiosis-solutions.co.jp; Tel.: +81-3-6275-0878

Supplementary Table S1. Diseases present among participants in the Shokuyo and NJ diet groups.

Disease name	Shokuyo diet group (*n* = 19)	NJ diet group (*n* = 50)
With some kind of disease	8 (42.1%)	31 (62.0%)
High blood pressure	3 (15.8%)	11 (22.0%)
Dyslipidemia	4 (21.1%)	8 (16.0%)
Bone and joint diseases	0 (0.0%)	6 (12.0%)
Hay fever	2 (10.5%)	4 (8.0%)
Dizziness	0 (0.0%)	3 (6.0%)
Obesity	0 (0.0%)	3 (6.0%)
Type 2 diabetes	0 (0.0%)	3 (6.0%)
Constipation	0 (0.0%)	3 (6.0%)
Insomnia	0 (0.0%)	3 (6.0%)
Allergic rhinitis	0 (0.0%)	2 (4.0%)
Type 1 diabetes	0 (0.0%)	2 (4.0%)
Spinal stenosis	0 (0.0%)	2 (4.0%)
Skin diseases	1 (5.3%)	1 (2.0%)
Periodontal disease	1 (5.3%)	1 (2.0%)
Cataract	1 (5.3%)	1 (2.0%)
Hashimoto's thyroiditis	1 (5.3%)	1 (2.0%)
Hypothyroidism	0 (0.0%)	2 (4.0%)
Mild cognitive impairment	0 (0.0%)	2 (4.0%)
Rheumatoid arthritis	0 (0.0%)	2 (4.0%)
Reflux esophagitis	0 (0.0%)	2 (4.0%)
Chronic kidney disease	0 (0.0%)	2 (4.0%)
Stomach ulcer	0 (0.0%)	2 (4.0%)
Graves' disease	0 (0.0%)	1 (2.0%)
Systemic lupus erythematosus	0 (0.0%)	1 (2.0%)
Dry mouth	0 (0.0%)	1 (2.0%)
Dry eye	0 (0.0%)	1 (2.0%)
Colon polyp	0 (0.0%)	1 (2.0%)
Back pain, joint pain	0 (0.0%)	1 (2.0%)
Breast cancer	0 (0.0%)	1 (2.0%)
Weight loss	1 (5.3%)	0 (0.0%)
Depression	1 (5.3%)	0 (0.0%)
Bipolar disorder	0 (0.0%)	1 (2.0%)
Fatigue and tiredness	0 (0.0%)	1 (2.0%)
Cold Sensitivity	0 (0.0%)	1 (2.0%)
Alcohol allergy	0 (0.0%)	1 (2.0%)
Gastritis	0 (0.0%)	1 (2.0%)
Tongue cancer	0 (0.0%)	1 (2.0%)
Other musculoskeletal and connective tissue diseases	0 (0.0%)	1 (2.0%)
Uterine fibroids	0 (0.0%)	1 (2.0%)
Glaucoma	0 (0.0%)	1 (2.0%)
Angina pectoris	0 (0.0%)	1 (2.0%)
Eosinophilic sinusitis	0 (0.0%)	1 (2.0%)
Thyroid cancer	0 (0.0%)	1 (2.0%)

Number of patients within the group and percentage.

Supplementary Table S2. Comparison between the Shokuyo diet group and the NJ diet H subgroup.

	**Shokuyo diet (*n* = 19)**	**NJ diet H (*n* = 19)**	***p*-value**
Age (year)	70.4 ± 4.1	67.1 ± 4.5	0.035
Height (cm)	155.0 ± 4.3	157.1 ± 5.4	0.178
Weight (kg)	48.4±6.8	55.3 ± 6.5	0.008
BMI (kg/m^2)	20.0±2.6	22.4 ± 2.7	0.013
α-diversity indices:			
Shannon index	2.86 ± 0.24	2.79 ± 0.17	0.506
Simpson index	0.91 ± 0.02	0.90 ± 0.03	1.000
Number of taxa	54.00 ± 8.63	49.32 ± 9.06	0.136
Pielou index	0.72 ± 0.04	0.72 ± 0.04	0.954

Data are presented as the mean ± standard deviation. *p*-values obtained from the Wilcoxon rank-sum test. BMI: body mass index. Statistical significance was set at $p < 0.05$.

Supplementary Table S3. Comparison between the Shokuyo diet group and the NJ diet UH subgroup.

	**Shokuyo diet (*n* = 19)**	**NJ diet UH (*n* = 31)**	***p*-value**
Age (year)	70.4 ± 4.1	69.4 ± 6.0	0.502
Height (cm)	155.0 ± 4.3	155.1 ± 5.6	0.645
Weight (kg)	48.4 ± 6.8	53.6 ± 7.8	0.023
BMI (kg/m^2)	20.0 ± 2.6	22.2 ± 3.0	0.018
α-diversity indices:			
Shannon index	2.86 ± 0.24	2.93 ± 0.21	0.218
Simpson index	0.91 ± 0.02	0.92 ± 0.02	0.176
Number of taxa	54.00 ± 8.63	56.26 ± 10.70	0.653
Pielou index	0.72 ± 0.04	0.73 ± 0.04	0.283

Data are presented as the mean ± standard deviation. *p*-values obtained from the Wilcoxon rank-sum test. BMI: body mass index. Statistical significance was set at $p < 0.05$.

Supplementary Table S4. Effect sizes, relative abundance, and detection rates of each taxon in the ALDEx2 analysis between the Shokuyo diet group and the NJ diet UH subgroup.

Taxa (genus level)	Effect size	Shokuyo diet group			NJ diet UH subgroup		
		Relative abundance (%)		Detection rate (%)	Relative abundance (%)		Detection rate (%)
		Mean ± SD	Median		Mean ± SD	Median	
Kineothrix	0.66	0.67 ± 0.75	0.35	84.2	0.09 ± 0.15	0.00	48.4
Anaerostipes	0.62	1.73 ± 1.75	1.40	100.0	0.68 ± 0.63	0.55	100.0
Coprococcus	0.56	1.21 ± 1.71	0.39	78.9	0.39 ± 0.91	0.00	38.7
Enterobacter	0.48	0.12 ± 0.18	0.00	42.1	0.01 ± 0.04	0.00	6.5
Clostridium_sensu_stricto	0.47	0.43 ± 0.52	0.11	73.7	0.14 ± 0.36	0.00	48.4
Agathobaculum	0.37	0.63 ± 0.48	0.53	84.2	0.25 ± 0.3	0.13	67.7
Faecalibacillus	0.31	1.58 ± 1.94	0.64	73.7	0.76 ± 1.09	0.00	45.2
Blautia	0.31	6.28 ± 2.26	6.79	100.0	5.45 ± 1.86	5.08	100.0
Veillonella	0.28	0.37 ± 0.85	0.04	78.9	0.24 ± 0.44	0.00	48.4
Turicibacter	0.27	0.16 ± 0.23	0.03	57.9	0.08 ± 0.17	0.00	41.9
Unclassified	0.26	14.94 ± 6.39	14.25	100.0	13.91 ± 8.65	11.48	100.0
Adlercreutzia	0.26	0.1 ± 0.08	0.12	73.7	0.11 ± 0.21	0.00	48.4
Senegalimassilia	0.26	0.11 ± 0.17	0.00	42.1	0.06 ± 0.14	0.00	22.6
Prevotella	0.22	7.81 ± 10.3	0.00	47.4	3.43 ± 7.77	0.00	35.5
Parasutterella	0.21	0.15 ± 0.32	0.01	57.9	0.17 ± 0.42	0.00	29.0
Lawsonibacter	-0.20	0.02 ± 0.03	0.00	42.1	0.06 ± 0.12	0.01	51.6
Akkermansia	-0.20	0.26 ± 0.48	0.03	57.9	1.57 ± 2.91	0.15	54.8
Phocaeicola	-0.21	7.69 ± 6.15	5.55	100.0	10.68 ± 6.32	9.93	96.8
Eubacterium	-0.21	0 ± 0	0.00	0.0	0.01 ± 0.03	0.00	29.0
Alistipes	-0.22	0.92 ± 0.91	0.66	78.9	1.93 ± 2.09	1.20	93.5
Fusobacterium	-0.23	0.66 ± 2.87	0.00	15.8	1.29 ± 3.35	0.00	38.7
Anaerotignum	-0.23	0.09 ± 0.1	0.07	78.9	0.22 ± 0.26	0.15	90.3
Negativibacillus	-0.24	0.03 ± 0.06	0.00	42.1	0.09 ± 0.15	0.03	61.3
Coprobacillus	-0.25	0 ± 0	0.00	0.0	0.1 ± 0.42	0.00	29.0
Sellimonas	-0.27	0.01 ± 0.03	0.00	15.8	0.1 ± 0.18	0.00	38.7
Ihubacter	-0.28	0 ± 0.01	0.00	10.5	0.02 ± 0.03	0.00	35.5
Bilophila	-0.28	0.06 ± 0.07	0.04	63.2	0.16 ± 0.15	0.16	80.6
Bacteroides	-0.30	5.73 ± 5.3	3.95	100.0	7.82 ± 4.46	7.30	100.0
Lactobacillus	-0.34	0.01 ± 0.04	0.00	15.8	0.14 ± 0.45	0.02	54.8
Erysipelatoclostridium	-0.42	0.05 ± 0.14	0.00	21.1	0.14 ± 0.19	0.05	58.1
Dysosmobacter	-0.44	0.1 ± 0.11	0.04	84.2	0.31 ± 0.28	0.24	90.3
Escherichia.Shigella	-0.49	0.95 ± 3.77	0.00	31.6	0.8 ± 1.42	0.08	71.0
Eggerthella	-0.56	0.04 ± 0.08	0.00	31.6	0.17 ± 0.21	0.11	77.4
Ruthenibacterium	-0.57	0.01 ± 0.02	0.00	36.8	0.16 ± 0.24	0.08	74.2
Enterocloster	-0.58	0.06 ± 0.14	0.00	36.8	0.24 ± 0.31	0.13	77.4
Flavonifractor	-0.62	0.06 ± 0.1	0.00	42.1	0.22 ± 0.25	0.13	87.1

Data with absolute effect sizes of 0.2 or higher are presented.

Supplementary Table S5. Effect sizes, relative abundance, and detection rates of each taxon in the ALDEx2 analysis between the Shokuyo diet group and the NJ diet H subgroup.

Taxa (genus level)	Effect size	Shokuyo diet group			NJ diet H subgroup		
		Relative abundance (%)		Detection rate (%)	Relative abundance (%)		Detection rate (%)
		Mean ± SD	Median		Mean ± SD	Median	
Coprococcus	0.56	1.21 ± 1.71	0.39	78.9	0.11 ± 0.15	0.00	47.4
Unclassified	0.49	14.94 ± 6.39	14.25	100.0	11.64 ± 5.45	11.70	100.0
Adlercreutzia	0.47	0.1 ± 0.08	0.12	73.7	0.03 ± 0.05	0.00	42.1
Kineothrix	0.43	0.67 ± 0.75	0.35	84.2	0.23 ± 0.43	0.00	47.4
Blautia	0.42	6.28 ± 2.26	6.79	100.0	5.55 ± 2.62	5.30	100.0
Enterobacter	0.34	0.12 ± 0.18	0.00	42.1	0.13 ± 0.5	0.00	10.5
Senegalimassilia	0.29	0.11 ± 0.17	0.00	42.1	0.06 ± 0.13	0.00	15.8
Agathobaculum	0.29	0.63 ± 0.48	0.53	84.2	0.33 ± 0.34	0.24	73.7
Megasphaera	0.26	0.28 ± 0.79	0.00	47.4	0.2 ± 0.57	0.00	21.1
Intestinimonas	0.26	0.06 ± 0.05	0.06	78.9	0.04 ± 0.07	0.00	42.1
Turicibacter	0.24	0.16 ± 0.23	0.03	57.9	0.05 ± 0.1	0.00	42.1
Oscillibacter	0.23	0.18 ± 0.32	0.03	63.2	0.13 ± 0.38	0.00	42.1
Bacillus	0.22	0.05 ± 0.09	0.00	47.4	0.02 ± 0.03	0.00	31.6
Raoultibacter	0.21	0.04 ± 0.06	0.00	42.1	0.01 ± 0.03	0.00	15.8
Faecalibacillus	0.21	1.58 ± 1.94	0.64	73.7	1.08 ± 1.68	0.33	52.6
Klebsiella	0.20	1.22 ± 2.95	0.00	42.1	0.95 ± 2.32	0.00	26.3
Longicatena	-0.21	0 ± 0	0.00	5.3	0.02 ± 0.04	0.00	21.1
Lacticaseibacillus	-0.21	0.01 ± 0.03	0.00	5.3	0.06 ± 0.13	0.00	21.1
Roseburia	-0.21	1.62 ± 1.42	1.35	94.7	2.74 ± 1.99	2.82	94.7
Parabacteroides	-0.22	1.06 ± 0.79	0.89	100.0	2.13 ± 1.86	1.70	89.5
Bilophila	-0.24	0.06 ± 0.07	0.04	63.2	0.16 ± 0.18	0.13	73.7
Enterococcus	-0.25	0.29 ± 1.27	0.00	15.8	1.12 ± 3.89	0.00	31.6
Dysosmobacter	-0.27	0.1 ± 0.11	0.04	84.2	0.23 ± 0.26	0.11	89.5
Escherichia.Shigella	-0.29	0.95 ± 3.77	0.00	31.6	1.37 ± 3.61	0.04	57.9
Flavonifractor	-0.33	0.06 ± 0.1	0.00	42.1	0.14 ± 0.18	0.09	68.4
Colidextribacter	-0.33	0 ± 0.01	0.00	5.3	0.02 ± 0.03	0.00	31.6
Sellimonas	-0.35	0.01 ± 0.03	0.00	15.8	0.11 ± 0.26	0.00	47.4
Lawsonibacter	-0.36	0.02 ± 0.03	0.00	42.1	0.06 ± 0.07	0.04	68.4
Erysipelatoclostridium	-0.37	0.05 ± 0.14	0.00	21.1	0.25 ± 0.74	0.03	57.9
Eggerthella	-0.44	0.04 ± 0.08	0.00	31.6	0.16 ± 0.24	0.11	68.4

Data with absolute effect sizes of 0.2 or higher are presented.

Supplementary Table S6. Relative abundance and detection rate of each intestinal bacterial taxon in the Shokuyo diet group (n = 19).

Taxa (genus level)	Relative abundance (%)		Detection rate (%)
	Mean ± SD	Median	
Adlercreutzia	0.10 ± 0.08	0.12	73.7
Agathobacter	2.85 ± 2.76	2.21	68.4
Agathobaculum	0.63 ± 0.48	0.53	84.2
Akkermansia	0.26 ± 0.48	0.03	57.9
Alistipes	0.92 ± 0.91	0.66	78.9
Allisonella	0.01 ± 0.02	0.00	36.8
Anaerobutyricum	0.51 ± 0.31	0.47	89.5
Anaerostipes	1.73 ± 1.75	1.40	100.0
Anaerotignum	0.09 ± 0.10	0.07	78.9
Bacillus	0.05 ± 0.09	0.00	47.4
Bacteroides	5.73 ± 5.30	3.95	100.0
Barnesiella	0.31 ± 0.52	0.00	47.4
Bifidobacterium	5.10 ± 4.56	3.87	94.7
Bilophila	0.06 ± 0.07	0.04	63.2
Blautia	6.28 ± 2.26	6.79	100.0
Butyricicoccus	0.03 ± 0.07	0.00	26.3
Butyricimonas	0.12 ± 0.14	0.03	57.9
*Clostridium*_IV	0.19 ± 0.43	0.00	42.1
Clostridium_sensu_stricto	0.43 ± 0.52	0.11	73.7
*Clostridium*_XlVa	0.04 ± 0.09	0.00	36.8
*Clostridium*_XlVb	0.13 ± 0.44	0.00	31.6
*Clostridium*_XVIII	0.03 ± 0.05	0.00	36.8
Collinsella	1.39 ± 1.12	1.02	89.5
Coprobacter	0.05 ± 0.14	0.00	36.8
Coprococcus	1.21 ± 1.71	0.39	78.9
Dialister	0.87 ± 1.21	0.00	47.4
Dorea	0.78 ± 0.61	1.00	84.2
Duodenibacillus	0.07 ± 0.21	0.00	26.3
Dysosmobacter	0.10 ± 0.11	0.04	84.2
Eggerthella	0.04 ± 0.08	0.00	31.6
Enterobacter	0.12 ± 0.18	0.00	42.1
Enterocloster	0.06 ± 0.14	0.00	36.8
Escherichia.Shigella	0.95 ± 3.77	0.00	31.6
Faecalibacillus	1.58 ± 1.94	0.64	73.7
Faecalibacterium	9.66 ± 4.97	10.76	94.7
Flavonifractor	0.06 ± 0.10	0.00	42.1
Flintibacter	0.08 ± 0.09	0.06	78.9
Fusicatenibacter	3.93 ± 3.65	3.57	100.0
Gemmiger	2.16 ± 1.44	1.89	89.5
Gordonibacter	0.01 ± 0.01	0.00	26.3
Haemophilus	0.16 ± 0.38	0.02	63.2
Holdemanella	0.82 ± 1.37	0.00	36.8
Holdemania	0.02 ± 0.04	0.00	47.4
Intestinimonas	0.06 ± 0.05	0.06	78.9
Kineothrix	0.67 ± 0.75	0.35	84.2
Klebsiella	1.22 ± 2.95	0.00	42.1
Lachnospira	1.77 ± 1.18	1.85	89.5
Lawsonibacter	0.02 ± 0.03	0.00	42.1

Limosilactobacillus	0.01 ± 0.03	0.00	31.6
Massilimicrobiota	0.03 ± 0.05	0.00	42.1
Mediterraneibacter	0.54 ± 0.48	0.59	73.7
Megasphaera	0.28 ± 0.79	0.00	47.4
Merdimonas	0.02 ± 0.05	0.00	31.6
Monoglobus	0.03 ± 0.07	0.00	26.3
Negativibacillus	0.03 ± 0.06	0.00	42.1
Neglecta	0.10 ± 0.13	0.05	68.4
Odoribacter	0.20 ± 0.23	0.20	84.2
Oscillibacter	0.18 ± 0.32	0.03	63.2
Parabacteroides	1.06 ± 0.79	0.89	100.0
Paraprevotella	0.34 ± 0.62	0.00	47.4
Parasutterella	0.15 ± 0.32	0.01	57.9
Phascolarctobacterium	0.66 ± 0.73	0.31	52.6
Phocaeicola	7.69 ± 6.15	5.55	100.0
Prevotella	7.81 ± 10.3	0.00	47.4
Raoultibacter	0.04 ± 0.06	0.00	42.1
Romboutsia	0.52 ± 1.81	0.04	68.4
Roseburia	1.62 ± 1.42	1.35	94.7
Rothia	0.01 ± 0.02	0.00	26.3
Ruminococcus	3.15 ± 2.53	3.24	84.2
Ruthenibacterium	0.01 ± 0.02	0.00	36.8
Schaalia	0.01 ± 0.02	0.00	26.3
Senegalimassilia	0.11 ± 0.17	0.00	42.1
Slackia	0.10 ± 0.17	0.00	31.6
Streptococcus	1.88 ± 3.67	0.63	94.7
Sutterella	0.49 ± 0.72	0.24	68.4
Turicibacter	0.16 ± 0.23	0.03	57.9
Veillonella	0.37 ± 0.85	0.04	78.9
Unclassified	14.94 ± 6.39	14.25	100.0

Only taxa with a detection rate of 0.25% or higher are presented.

Supplementary Table S7. Duration of the Shokuyo diet.

Duration (year)	number of participants
1	2
4	2
5	1
6	1
7	1
10	1
12	2
15	3
20	3
30	1
40	1
48	1
